# Gum-based nanocomposites for the removal of metals and dyes from waste water

**DOI:** 10.1007/s11356-023-29389-6

**Published:** 2023-09-07

**Authors:** Muhammad Usman, Muhammad Babar Taj, Sónia Alexandra Correia Carabineiro

**Affiliations:** 1 Institute of Chemistry, The Islamia University of Bahawalpur, Bahawalpur, 63100 Pakistan; 2 LAQV-REQUIMTE, Department of Chemistry, NOVA School of Science and Technology, Universidade NOVA de Lisboa, 2829-516 Caparica, Portugal

**Keywords:** Gum-based nanomaterials, Waste water treatment, Guar gum, Xanthan gum, Adsorption, Degradation

## Abstract

The importance of water for all living organisms is unquestionable and protecting its sources is crucial. In order to reduce water contaminants, like toxic metals and organic dyes, researchers are exploring different techniques, such as adsorption, photocatalytic degradation, and electrolysis. Novel materials are also being sought. In particular, biopolymers like guar gum and xanthan gum, that are eco-friendly, non-toxic, reusable, abundant and cost-effective, have enormous potential. Gum-based nanocomposites can be prepared and used for removing heavy metals and colored dyes by adsorption and degradation, respectively. This review explains the significance of gum-based nanomaterials in waste water treatment, including preparative steps, characterization techniques, kinetics models, and the degradation and adsorption mechanisms involved.

## Introduction

Water is essential for the survival of all living beings in the world (Khullar and Singh 2022). However, the availability of safe and accessible drinking water sources is a significant challenge that affects mankind. The degradation of water quality due to over-industrialization and urbanization is an important issue that requires urgent attention, in order to sustain the planet, its inhabitants, and the future generations.

Freshwater resources are scarce and their quality is being deteriorated, due to several contaminants and pollutants, like heavy metals and organic and inorganic dyes, which are dangerous and toxic. The primary source of these pollutants is untreated waste water from several industries, such as paper, leather, petrochemical, textile, plastic, cosmetics, and others (Zaman et al. 2020).

Water pollution also harms aquatic species, as the colored dyes reflect sunlight back into the water, blocking its penetration (Gupta 2016). Therefore, it is mandatory that immediate actions are taken to overcome water pollution and ensure the sustainability of the water resources.

The discharge of dyes from various sources is a significant concern, with the levels of chemical oxygen demand (>150 ppm), total organic carbon (>2900 mg/L), and biological oxygen demand (>80 mg/L) exceeding the acceptable limits in many water sources (Larbi et al. 2019). This contamination has negative effects on the aquatic populations, resulting in mutagenic and carcinogenic effects that can lead to severe diseases in the human nervous system, digestive system, kidneys, and liver. Even a small amount of organic dyes (1 × 10^−3^ mg/L) can be harmful to both animals and humans (Tkaczyk et al. 2020).

In addition to dyes, heavy metals are also a growing problem in water released from industries, such as fertilizers, pesticides, refineries, leather, and mining. Heavy metals, like arsenic, selenium, zinc, chromium, nickel, cobalt, cadmium, mercury, lead, and copper are harmful to living organisms even in small quantities (Amjad et al. 2020). The accumulation of these pollutants and contaminants in water turns it undrinkable and can cause serious health problems to living organisms (Liu et al. 2015).

**Fig. 1:**
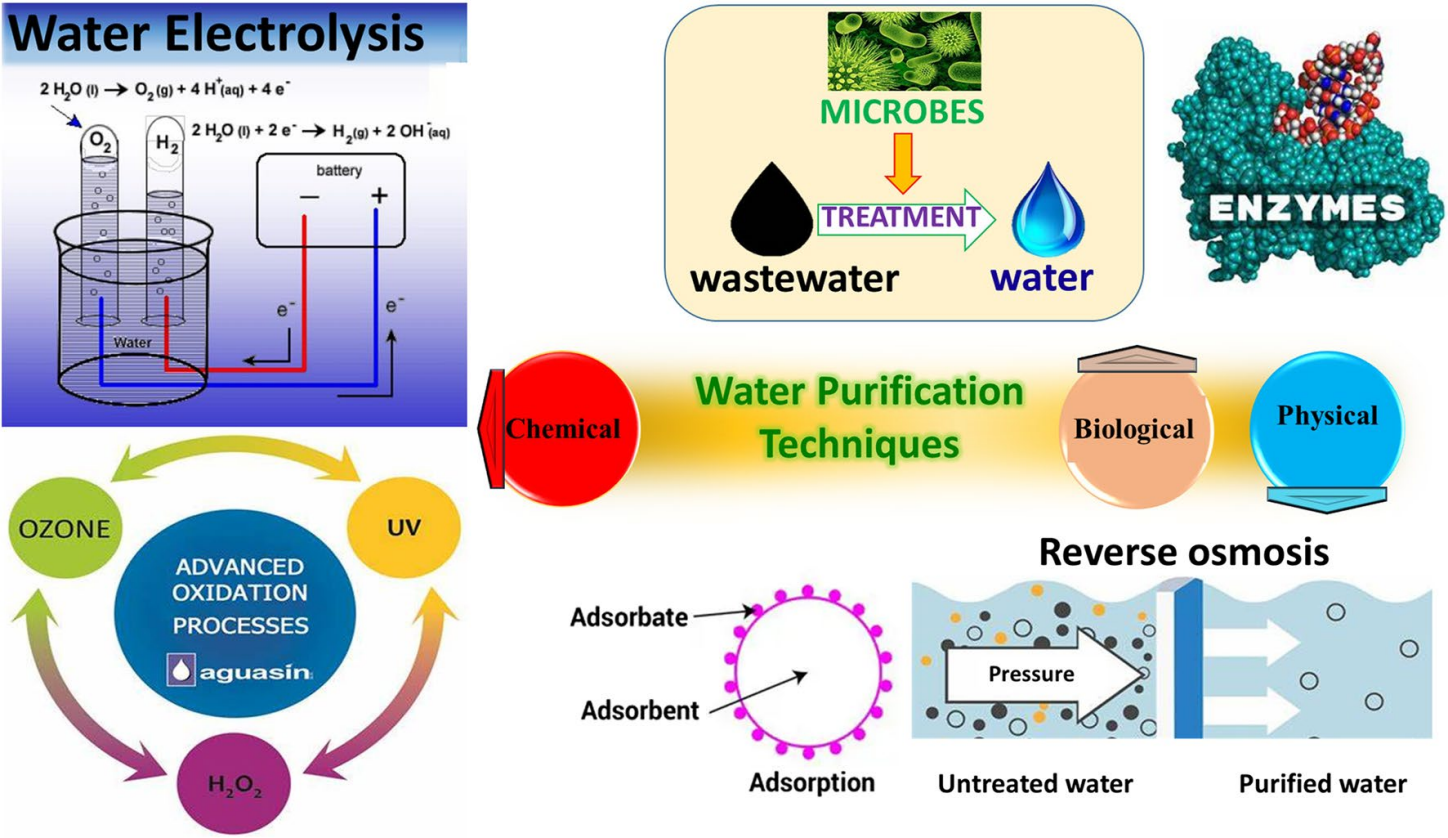
Water purification techniques

Due to these dangers, significant efforts are being made, in recent years, to find eco-friendly, affordable, and efficient processes to remove heavy metals and dyes from water. A variety of research is being conducted, using typical removal methods, such as coagulation, membrane filtration, ozonation, adsorption, flocculation, electrochemical techniques, reverse osmosis, biological methods, electrolysis, advanced oxidation processes, ultrafiltration, and sedimentation for waste water treatment, as shown in Fig. 1 (Saya et al. 2021; Morin-Crini et al. 2022).

Most of the methods mentioned in the previous studies have some limitations, like restricted removal efficiency, expensiveness, difficulty in processing, and the generation of secondary contaminants as by-products (Katheresan et al. 2018). Among all these techniques, adsorption is very popular, due to its many advantages, such as being cheaper, easier to handle, having great removal efficiency, low energy consumption, eco-friendliness, and a smart design (Qasem et al. 2021). In addition, photocatalytic degradation, combined with adsorption, is a promising strategy for the degradation of organic and inorganic dyes (Zaman et al. 2020).

Moreover, researchers aim to develop materials with a large surface area to enhance adsorption and improve degradation (Nasrollahzadeh et al. 2021). Several adsorbent materials, like zeolites (Maharana and Sen 2021), CNTs (Sajid et al. 2022), metal-organic frameworks (Adegoke et al. 2020), and activated carbons (Mendes Ferreira and Melo 2021) have been extensively studied. Natural polysaccharides are promising alternatives, as they are non-toxic, inexpensive, readily available, and abundant in nature, making them suitable candidates for adsorption (Mignon et al. 2019; Ge et al. 2023; Jalili et al. 2023; Mandal et al. 2023). The high number of active groups in the backbone of polysaccharides allows them to be easily modified to improve their adsorption capacity (Wei et al. 2017). Additionally, they can be incorporated in various ways with different materials, resulting in the development of new samples with improved properties (Saya et al. 2020).

Also, novel gum-based nanocomposites have been developed for the removal of heavy metal particles, organic, and inorganic dyes from contaminated water. This review highlights the working mechanism of the gum nanocomposites as adsorbents and their effectiveness in removing colored dyes and toxic metal ions. The review emphasizes the significant role of gum-based nanocomposites in efficiently removing pollutants from water and provides insights for researchers in their future investigations.

## Structure of guar gum and its applications

**Fig. 2:**
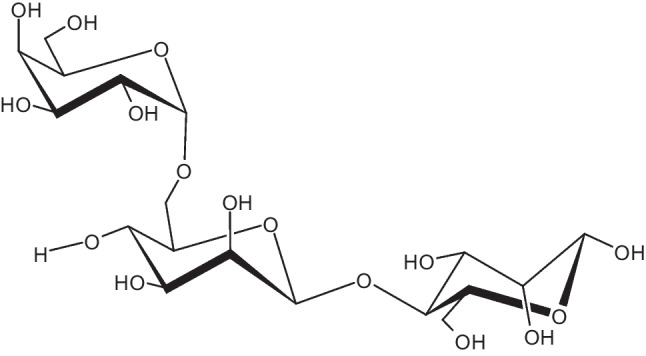
Structure of guar gum

Guar gum (GG) is a natural and inexpensive source of galactomannan, with a structure consisting of alternate linear chains of galactose and mannose portions, as shown in Fig. 2. The ratio of mannose to galactose in GG is typically between 1.6:1 and 1.8:1 (Adimule et al. 2022). GG has a high molecular weight, ranging from 1 × 10^6^ to 3 × 10^6^ g/mol and is very soluble in water (Zaeim et al. 2022). Its composition includes 0.67% N, 1.05% ash, 3.5–4% protein, 7.75% water-insoluble portion, 0.006% phosphorous, 88.5% water-soluble polysaccharides, and 1.5% alcohol-soluble compounds. GG also has interesting chemical characteristics, like chain length, steric obstacles, substituent groups, etc. It is insoluble in organic solvents but forms a highly viscous mixture in water, even at low concentrations, due to the presence of a galactose side chain that rapidly interlinks with water molecules. The viscosity of GG (3000-6000cP) is highly dependent on pH, concentration, impurities, and temperature. Moreover, the addition of GG can form gel-like materials (Thombare et al. 2016; Soltani et al. 2021). GG has a high number of cis O–H groups in its backbone, as shown in Fig. 2, which facilitates its attachment to organic and hydrated minerals by hydrogen bonding. GG is also an effective coagulant for inorganic systems that resemble clay and for carboxyl and hydroxyl-containing organic systems (Thombare et al. 2016).

GG can be extracted from the endosperm of the seeds of *Cyamopsis tetragonoloba*, a water shortage bearing plant belonging to the *Leguminosae* family and commonly known as “cluster bean” (Teja et al. 2022). Various extraction methods can be employed, including mechanical stages, such as roasting, grinding, and sieving, followed by polishing (Mudgil et al. 2014; Manjunath et al. 2016). The seeds are then converted into powder and further processed to obtain the desired outcome.

**Fig. 3:**
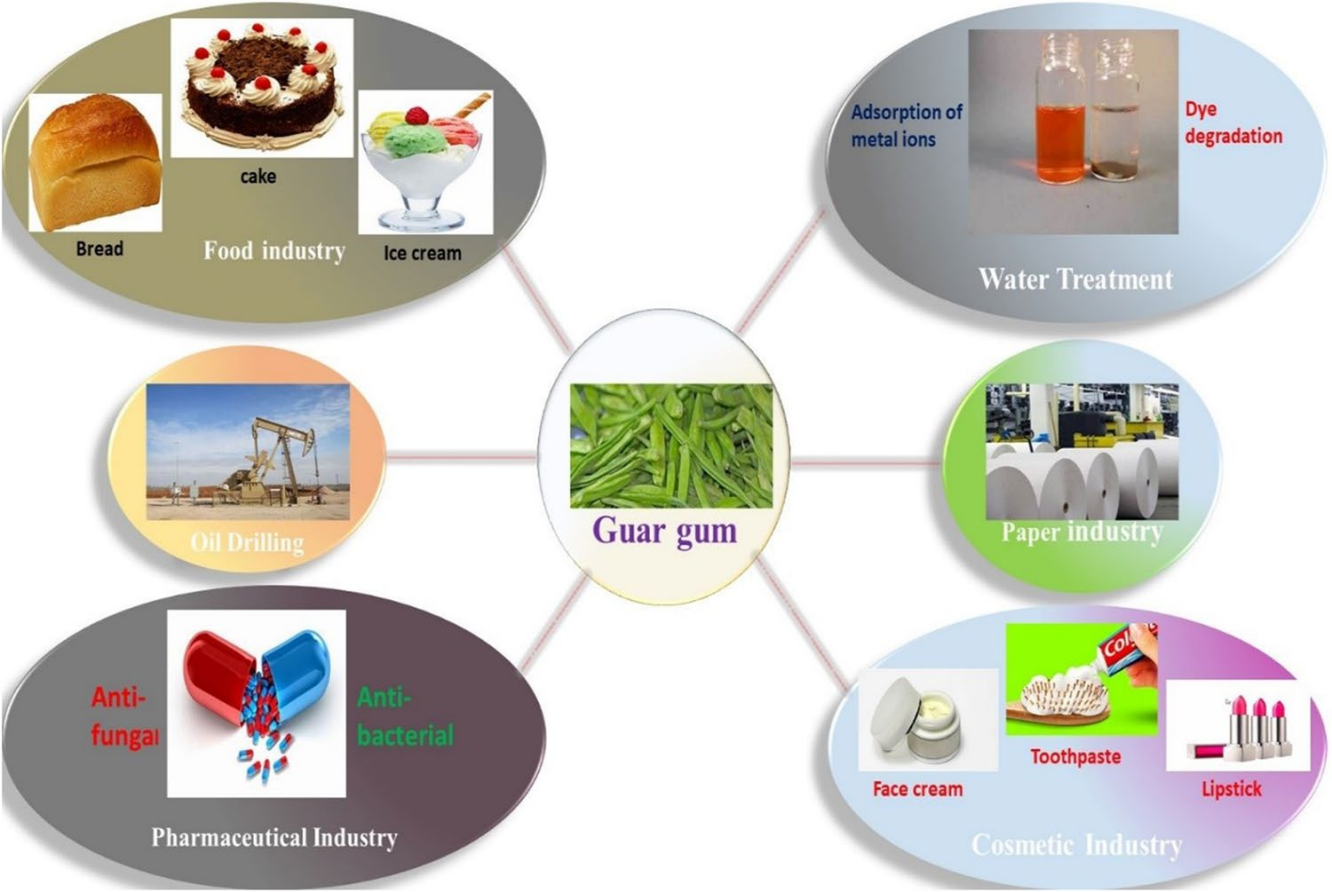
Applications of guar gum

GG and its derivatives find extensive applications in the pharmaceutical industry, where they serve as disintegrants and impart cohesive properties to drugs (Verma and Sharma 2021). GG also has remarkable potential in the cosmetic industry (Kumar et al. 2022a), owing to its high solubility in water, non-toxicity, affordability, easy availability, pH resistance, and ability to form colloidal suspensions (Sharma et al. 2018). GG is also utilized in food industry (Jiang et al. 2022), due to its low evaporation, high water capability, and modified freezing rate (Mudgil et al. 2014). It is also therapeutically used for its hypoglycemic (Xu et al. 2022), antimicrobial (Saurabh et al. 2018), appetite suppressant (Rao 2016), bulk-forming laxative (Purohit and Mishra 2017), hypolipidemic (Setayesh et al. 2022), antiproliferative (Ghosh et al. 2018), antianemic (Ganie et al. 2021), anti-inflammatory effects in colitis (Jhundoo et al. 2021; Hu et al. 2022), and Crohn’s disease (Varma et al. 2016). It is also used as a food additive in various dietary products such as cakes, tomato ketchup, pasta, ice cream, and in personal care products, like shaving creams, lotions, face creams, and toothpaste (Mudgil et al. 2014; Behera et al. 2022; Treudler and Simon 2022). Figure 3 depicts the different applications of GG in several fields.

Although GG has a wide range of applications, its use is limited in some fields. Its modification can be achieved by introduction of different substituents in its backbone or embedding it with other materials to form semi-synthetic or nanomaterials. However, these modified materials may retain their inherent features, which can restrict their applications in certain areas (Hongbo et al. 2013; Kumar et al. 2015).

One of the most significant applications of GG-based nanocomposites is in waste water treatment (Ahmad et al. 2022), where they play an efficient role in providing solutions to the problems of pure water supply (Dalei and Das 2022). These nanocomposites are often used as hybrid hydrogels, that have a spongy structure and are favorable adsorption materials (Jing et al. 2013). The embedding of hydrogels with natural materials decreases the cost and gives materials for specific needs and great efficiency. Additionally, hydrogels can be reused because of their good mechanical strength and unchanged nature during stirring. These GG-based nanocomposites exhibit adsorptive behavior towards toxic metal ions, like lead, chromium, cadmium, zinc, astatine, and others, as well as selective adsorption of dyes (cationic and anionic) depending on the morphological and chemical parameters (Saya et al. 2021). Some metal-grafted composites also exhibit photocatalytic degradation.

## Adsorption studies

Adsorption occurs on surfaces of some materials and is often described as the buildup of an excessive amount of an ingredient called an adsorbent on the outer surface of adsorbate, which is caused by the unequal surface residual forces found on the surface (Ighalo et al. 2022). It involves various interacting forces, like van der Waals, n-n interaction, hydrogen bonding, etc. The adsorption process plays a key role in waste water treatment (Chakraborty et al. 2022), due to its special features like cost effectiveness, efficient removal, eco-friendliness, and simplicity (Gautam et al. 2020). A large number of adsorbent materials can be obtained with good adsorption capacity and removal efficiency.

Different parameters can have a significant effect on the removal efficiency, such as time of contact, pH, quantity of adsorbent and adsorbate, temperature, etc. The effect of these parameters can be investigated by changing one parameter while keeping all others constant, as different parameters have different effects. Since adsorption is a heat-evolving process (exothermic), the equilibrium is disturbed by increasing temperature and thus the desorption process increases. Similarly, by raising the quantity of adsorbent and the time of contact, the adsorption process increases but only up to a certain point. After that, there will be no more effect because, after the establishment of the equilibrium, the surface is fully saturated and is unable to further accept any more adsorbate.

Like other parameters, pH also influences the adsorption ability of the adsorbent, which is described by the zeta potential. As the pH alters, it changes the charge of the adsorbent surface and also has an impact on the interaction of the nanocomposite with pollutants. In order to analyze the pH effect, adsorption on the dye is observed on the surface when all other parameters are kept constant, like temperature, time for contact and initial concentration, and the impact of pH is analyzed at regular intervals of time. This is consistent with the adsorption findings, which show efficient adsorption at alkaline pH around 5.1–7.6 for crystal violet (cationic dyes) and metal ions. Moreover, sometimes, by changing pH, protonation, and deprotonation of functional groups occur, which affect the interacting ability of the adsorbent as well. For example, in the case of an anionically developed GG/SiO_2_ nanocomposites, in which deprotonation of –COOH group of hydrolyzed polyacrylamide is carried out in a basic medium, this results in a negatively charged surface that interacts with cationic or metal ions instead of anionic particles (Patra et al. 2017).

When adsorbent and adsorbate come into contact with each other, adsorption occurs. But at the same time, desorption also starts, but its value is small in the beginning. As time progresses, desorption also increases and equilibrium between both processes (adsorption and desorption) takes place. Depending on many factors, several kinetic models can be used to predict the rate and feasibility of the adsorption process and mechanism.

To examine the kinetics models, the concentration of dye or metals can be calculated by ultraviolet-visible (UV-Vis) spectrophotometry at comparable *λ*_max_. The pseudo-first-order kinetics model of Lagergren only gives the physical relationship of adsorbate and adsorbent (González-Ipia et al. 2020), whereas the second order model explains chemisorption. While the intraparticle diffusion model expects adsorption, which involves three steps, one or a mixture of these steps can be the rate-determining steps: (1) movement of mass through the outer bordered layer film of liquid; (2) fast adsorption of adsorbate on an adsorbent surface by chemisorption or physisorption; (3) diffusion of adsorbate on an adsorbent active spot, which is commonly the rate-determining stage (Wu et al. 2009). In the model of liquid diffusion, the rate controlling stage is the diffusion phase of liquid film in spite of intraparticle diffusion.

Similarly, a detailed investigation of adsorption isotherm models helps in the investigation of the interaction of the adsorbent with dyes/metals or metal ions (adsorbate). In order to obtain the adsorption isotherm, after a fixed duration from the concentration at the initial point, the equilibrium concentration of the adsorbate solution can be determined. The Langmuir adsorption isotherm model proposes a monolayer of adsorbate (metal ion/dyes) on the adsorbent surface at a specific number of active spots with negligible steric hindrance effect and interaction molecules that are adsorbed (Zafar et al. 2022). So, after the complete coverage of the surface, no more adsorption occurs. Freundlich isotherm also gives monolayer predictions with no restriction on the multilayer of adsorption (Chen et al. 2022a). Dubinin-Radushkevich’s model explains the formation of multilayer physical adsorption through the interaction of van der Waal’s forces. This model is useful in the separation of physical and chemical adsorption mechanisms (Al Jaberi et al. 2020). The sips model explains the combination of the Freundlich and Langmuir isotherms in the same manner and removes the restrictions of the Freundlich adsorption model (Chen et al. 2022a). Toth’s model also provides the best estimates on adsorption isotherms (Yeo et al. 2023). The whole data are included into specific equations of each adsorption isotherm model and the topmost-fitted models are chosen based on the regression coefficient (*R*^2^) for describing the mechanism of adsorption.

## Photocatalytic degradation studies

The mechanism of degradation explains how large dyes are broken down, resulting in small molecules like CO_2_, H_2_O, O_2_, etc., in the presence of light. The possibility and dye degradation depends on the band gap energy of the nanoparticles.

The photocatalytic degradation of dyes can be explained in two possible ways:

1. Photocatalytic degradation by synergistic adsorption. This involves the photocatalytic degradation of dyes when adsorption immediately occurs on the adsorbent surface, without the need of putting it in the dark (Chen et al. 2022b).
2. Equilibrium adsorption in the darkness by photocatalytic degradation. In this step, a dynamic equilibrium is formed between the adsorbed and the unabsorbed molecules of dye in the dark when the dye is adsorbed on the surface of nanoparticles. After a certain time, the solution is exposed to sunlight for further photocatalytic degradation of the dye (Guo et al. 2022).

When a dye is adsorbed on the surface of a nanomaterial, both degradation strategies yield a decrease in adsorption band intensities. When a dye is adsorbed on the surface of a nanoparticle, the dye removal process increases and the degradation time decreases. The percentage of degraded dye can be calculated by the following equation Eq. 1 (Alnassar et al. 2022).

1 $$\%\textrm{degradation}=\frac{C_0-{C}_t}{C_0}\times 100$$

where the concentration at the initial point is C_0_ and concentration at time t is C_t_.

The pseudo-first-order rate equation can be used to find the rate kinetics of photocatalytic degradation of dyes by Eq. 2 (Rauf et al. 2022).

2 $$\ln\ {\textrm{A}}_{\textrm{t}}/{\textrm{A}}_0={\textrm{k}}_{\textrm{app}}\textrm{t}$$

where k_app_ is the apparent rate constant. A_t_ and A_0_ are concentration after time t and before illumination, respectively.

## GG-based nanocomposites for the removal of dyes

There are several nanocomposites containing GG that can be used in the removal of dyes. Table 1 presents some examples. The most important ones and their major preparation methods are listed in the following sections.

**Table 1:** Several nanomaterials obtained by different synthesis methods used in the removal of diverse dyes from water

Nanomaterial	Synthesis	Dyes	Adsorption capacity (mg g^−1^)/removal efficiency (%)	References
Table GG/β-cyclodextrin	Sonication	Basic fuchsine	24 mg g^−1^	Zhang et al. (2012)
GG-MWCNT (GG multi-walled carbon nanotubes composite)	Sonication	Methylene blue	61.92 mg g^−1^	Yan et al. (2012)
GG/CTNC (guar gum-cerium (IV) tungstate nanocomposite)	Sol-gel	Methylene blue	99%	Dassanayake et al. (2019)
Modified GG-PAAm	Sonication and polymerization	Crystal violet and azure B	>85%	Hiremath and Vishalakshi (2015)
g-GG/ SiO_2_ (g. grafting)	Sol-gel	Reactive blue 4 and congo red	714.285 mg g^−1^ and 819.672 mg g^−1^	Guezzen et al. (2021)
GG/acrylic acid/nano clay	Microwave irradiation technique	Crystal violet	89%	Shruthi et al. (2016)
GG/SiO_2_	Sol-gel	Safranin and malachite green	281.69mg g^−1^ and 781.25mg g^−1^	Sharma et al. 2018
(Pc-cl-GG/SPION)	Polymerization/co-precipitation method	m-cresol and o-chlorophenol	176.1mg g^−1^ and 75.6 mg g^−1^	Sharma et al. (2017), Singh (2021)
Fe_3_O_4_-GG	Co-precipitation	Congo red		Dabi and Loonker (2021)
GG-g-(Am-co-SA-co-ASP)	Green polymerization	Methyl violet	53.28mg g^–1^	Singha et al. (2018)
Aminated GG/GO	Sonication	Rhodamine B	75%	Gopi et al. (2019)
GG-GH	Condensation and oxidation	Bromophenol blue	904.7 mg g^−1^	Duan et al. (2019)
AgNPs/GG/Poly (AA)	Cross linking and polymerization	Methylene blue	833.33mg g^−1^	Singh and Dhaliwal (2021)
(ZnO NPs/GG)	Co-precipitation	Reactive red and rhodamine B	70.44 mg g^−1^ and 72.96 mg g^−1^	Rezk et al. (2019)
P(PrAm/AMPS)	Free radical polymerization	Basic violet 1	96.25%	Karadağ et al. (2021)
GG/NiWO_4_	Sol-gel	Phloxine B and crystal violet	220.21mg g^−1^ and 170.42mg g^−1^	Hussain et al. (2021)
CMC/GG/CuO_2_	Simple stirring	Malachite green	92.4%	Naeini et al. (2021)
PF/Ag/PVDF-GG/UiO-66		CTAB and Congo red	89.4% and 92.3%	Mofradi et al. (2021)

### Nanocomposites made of natural and synthetic polymers

This kind of nanocomposites can be prepared in several ways, as will be explained below.

#### Co-precipitation method

**Fig. 4:**
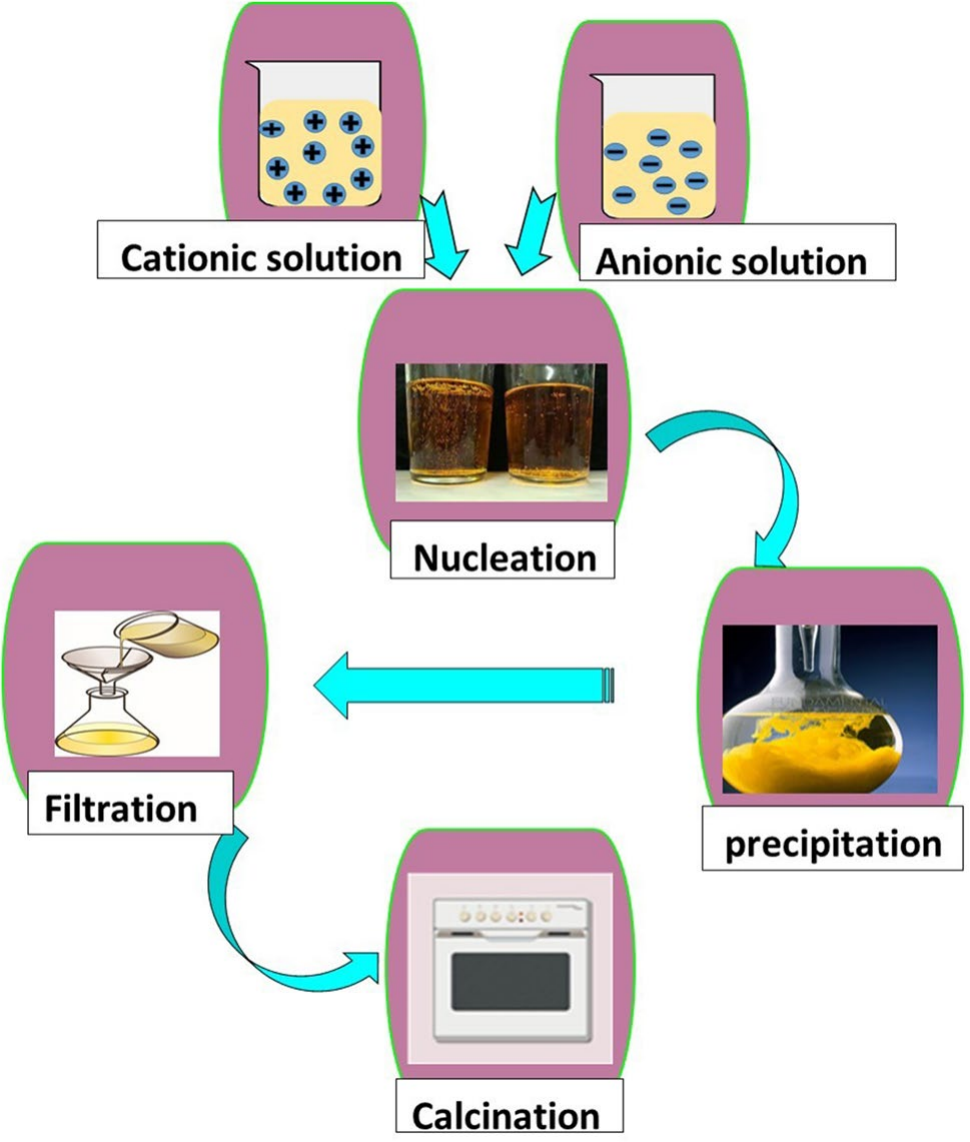
Co-precipitation method

The main intention of co-precipitation is the fabrication of a multicomponent material through the formation of an intermediate precipitate. Chemical homogeneity is achieved by calcination. Typically, the precipitation process involves mixing aqueous metal salts at an appropriate temperature in the presence of a base, which acts as the precipitating agent (Bajaj and Joshi 2021). This method is the most used for the synthesis of GG and other polysaccharides, due to its ability to be combined with other procedures, like polymerization, to form the desired products, as shown in Fig. 4. For example, pectin-crosslinked-guar gum/superparamagnetic iron oxide (Pc-cl-GG/SPION) nanomaterials can be synthesized using co-precipitation or polymerization (Sharma et al. 2017). The co-precipitation involves mixing equimolar concentrations of FeCl_3_ and FeCl_2_, followed by the addition of ammonium hydroxide to maintain a pH of 10. The resulting sol is then added to biopolymeric GG and pectin, and ammonium persulfate and methylene bisacrylamide are added as the initiator and crosslinker, respectively. The reaction mixture is stirred vigorously to form a hydrogel, which is effective in removing o-chlorophenol and m-cresol.

#### Solution intercalation method

The solution intercalation method is a versatile approach widely employed in materials science to introduce foreign species into the interlayer spaces of host materials, thereby enhancing their properties and functionalities. This method involves dispersing the desired species within a solvent, followed by immersing or infiltrating the host material into the solution. The intercalation process can be facilitated through various mechanisms, such as ion exchange, electrostatic interactions, or van der Waals forces, depending on the nature of the host and intercalant. Numerous studies have successfully utilized solution intercalation to modify the properties of various materials, including layered compounds, polymers, and nanomaterials (Rajapakse et al. 2021).

An eco-friendly and sustainable adsorbent can be formed by attaching GG onto an aluminum phyllosilicate clay, specifically bentonite, which mainly consists of montmorillonite. The resulting bio-nanomaterial shows remarkable efficiency in removing cationic dyes and heavy metal ions from waste water.

In a previous study, a nanocomposite was prepared using the solution intercalation method. Bentonite was added to double-distilled water, then mixed with a solution of GG, and subjected to ultrasonication (Ahmad and Mirza 2018).

#### Sonication

The process of sonication includes applying sound energy to a liquid containing particles using an ultrasonic bath or probe. To achieve optimal dispersion, researchers have discovered the benefits of combining sonication with mixing techniques, especially when employing probe sonicators. While the latter concentrate power into a smaller volume, their low shear forces may not ensure that all the polymer passes through this region, leading to incomplete dispersion. However, incorporating high-speed mixing alongside probe sonication has been reported as a relatively successful method for achieving nanoparticle dispersion. This combination approach effectively enhances dispersion, resulting in more consistent and uniform results (Taylor 2010).

GG is a versatile natural polysaccharide that can be combined with other natural polysaccharides to create nanocomposites with excellent adsorption properties. One such example is a nanocomposite made by embedding GG with β-cyclodextrin microspheres in various ratios, which exhibits an impressive adsorption potential of 24 mg/g towards basic fuchsine.

The nanostructured was prepared by converting the hydroxyl groups on GG into epoxy groups with epichlorohydrin. The resulting epoxidized GG was then allowed to interact with β-cyclodextrin: a mixture of epichlorohydrin, perchloric acid, ethanol, and distilled water was stirred with GG and toluene to obtain chloropropyl hydroxyl GG (cl-GG). Next, cl-GG was combined with β-cyclodextrin microspheres, sodium carbonate, and water. The pH was then lowered with hydrochloric acid, and the excess material was filtered and rinsed with *N,N*-dimethylformamide (DMF), ethanol, and acetone (Zhang et al. 2012).

#### Polymerization method

Polymerization plays a crucial role in the synthesis and fabrication of nanomaterials, enabling the creation of advanced materials with tailored properties. This process involves the reaction of monomers, small molecular units, to form long chains or networks, resulting in the formation of polymers at the nanoscale. The method offers precise control over the molecular structure, size, and morphology of the resulting nanomaterials, thereby influencing their physical, chemical, and mechanical characteristics. Various polymerization methods, such as radical polymerization, controlled/living polymerization, and ring-opening polymerization, have found extensive applications in the field. These techniques have facilitated the development of a wide range of nanomaterials for applications in diverse fields, including electronics, energy, medicine, and environmental remediation. Through polymerization, researchers can tailor nanomaterial properties to suit specific needs, leading to innovative solutions and advancements in various industries (Zhuang et al. 2015)

**Fig. 5:**
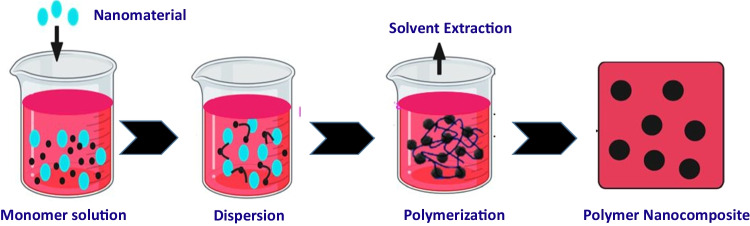
Polymerization technique

This method is schematically shown in Fig. 5. Grafting of GG and attachment of acrylamidosodiumpropanoate (ASP) by solution polymerization of acrylamide (Am) and sodium acrylate (SA) resulted in the synthesis of a sustainable GG-g-(Am-co-SA-co-ASP)/GGAMSAASP. The new GG-grafted terpolymer hydrogel nanocomposite showed adsorption through a different mechanism, specifically, the adsorption of methyl violet (MV) and Hg (II) by ligand selectivity (Singha et al. 2018).

Clinoptilolite, a natural zeolite, has been utilized to produce various biodegradable nanocomposites through the free radical polymerization method. Highly porous propenamide/2-acrylamido-2-methyl-1-propanesulfonic acid/guar gum/clinoptilolite biohybrid hydrogels P(PrAm/AMPS/CLP) were also prepared using a similar technique. The process involved using 0.25 mL of filtered water and 0.25 mL of a 2.0% CLP/water suspension instead of 0.5 mL of filtered water. The same procedure can be used to produce hybrid hydrogels with varying amounts of clinoptilolite (CLP) that were used to adsorb basic violet 1 (Karadağ et al. 2021).

#### Condensation method

This method enables the synthesis of nanostructured materials distinguished by their ultrafine grains, precise grain size distributions, well-defined grain boundaries, and exceptional resistance to grain growth. The condensation process affords a high level of control over various physical and chemical parameters, making it possible to achieve narrow particle and grain size distributions, ultimately enhancing the overall quality of the resulting materials (Suryanarayana and Prabhu 2007). Polymethyl methacrylate-modified dialdehyde guar gum containing hydrazide groups (GSA) was obtained by condensation between polyhydrazide and dialdehyde guar gum (DAG). Acrylic acid (AA) and methyl methacrylate (MMA) monomers were polymerized using potassium persulfate as the initiator to obtain poly(AA-co-MMA). *N,N′*-methylenebisacrylamide (MBA) utilized as the cross-linker by aqueous radical polymerization. Then poly(AA-co-MMA) was reacted with with hydrazine hydrate to obtain Poly(AA-co-MMASH). The functionalized GG nanocomposite showed excellent adsorption potential for methylene blue and malachite green from waste water (Wen et al. 2022).

#### Crosslinked method

A nano form catalyst based on an interpenetrating network (IPN) of polyacrylamide was developed using crosslinking and polymerization methods. The network structure of the nanocomposite was controlled by two cross-linkers. Upon saponification of the amide group, the resulting gel contained amino and carboxyl groups that exhibited significant adsorption towards cationic dyes such as azure B and crystal violet.

The guar gum–polyacrylamide (GG-PAAm) gel was prepared using free radical polymerization and crosslinking. GG was added to water. AAm and potassium persulfate (KPS) were dissolved in water and added to the GG solution. Then, methylene-bis-acrylamid (MBA) and GA were dissolved in water and mixed with the above solution. The resulting GG-PAAm gel was purified and subjected to alkaline hydrolysis modification (Hiremath and Vishalakshi 2015).

### GG-based nanocomposites of main group elements

Carbon-based nanomaterials, such as graphene oxide, activated carbon, and multi-walled carbon nanotubes (MWCNTs), were used to enhance the surface area and adsorption capacity of GG by grafting. For example, a nanocomposite of GG and activated carbon showed effective adsorption of anionic dyes such as Congo red (Yan et al. 2012).

#### Condensation and oxidation method

Galacylhydrazine grafted GG (GG-GH) was synthesized using condensation and oxidation methods and exhibited exceptional adsorption towards various dyes, such as bromophenol blue, methylene blue, methyl orange, and rhodamine B, with maximum adsorption capacities of 904.7, 1522.2, 868.83, and 1359.96 mg g^−1^, respectively.

The synthesis process involved dissolving aminated guar gum (AGG) in ethanol, then AGG and galacylhydrazine were heated, yielding a yellow precipitate (GG-GH) (Duan et al. 2019).

#### Hydrogel method

The hydrogel method stands as a versatile and promising technique in terms of biomaterials and tissue engineering. It revolves around the synthesis and manipulation of hydrogels, three-dimensional networks of crosslinked hydrophilic polymers that exhibit the remarkable capability of absorbing and retaining substantial amounts of water. These hydrogels possess a range of unique properties, including biocompatibility, tunable mechanical strength, and the ability to encapsulate cells or bioactive molecules (Utech and Boccaccini 2016).  GG-grafted with graphene oxide (AGG/GO) and carbon nanotubes (GG-CNT) demonstrated excellent adsorption towards cationic dyes (Gupta et al. 2020). To prepare the AGG/GO hydrogel, GO was sonicated in distilled water and then mixed with aminated GG. Next, a cross-linker solution of borax was added to the mixture, which was stirred until the hydrogel was formed. The hydrogel was then left without stirring to further solidify, and excess borax was removed by rinsing with water.

### GG-based nanocomposites of transition, lanthanide, and actinide elements

#### Co-precipitation

GG-based zinc oxide nanostructures, including nanocubes and nanoparticles, have excellent thermal stability. Among them, ZnO NPs/GG show very high photocatalytic degradation towards cationic and anionic dyes, such as rhodamine B (Rezk et al. 2019). They were synthesized by co-precipitation.

Magnetic nanoparticles of iron oxide (Fe_3_O_4_-GG) embedded in GG were very effective in removing various dyes, including Congo red, methylene blue, eriochrome black T, methyl blue, methyl orange, and malachite green. Congo red was found to have the highest efficiency, with a removal rate of up to 97% (Sahoo 2019; Dabi and Loonker 2021).

#### Sonication

GG-based tungsten oxide nanomaterials were prepared using a simple sonication method and demonstrated exceptional adsorption performance towards methylene blue, achieving up to 96% removal efficiency across varying concentrations of tungsten oxide.

To prepare the nanocomposites, polyvinyl alcohol (PVA) was dissolved in distilled water and mixed with a GG solution that was mixed with tetraethyl orthosilicate (TEOS) and sonicated. The composite was obtained after drying at room temperature in a dust-free environment (Hussain et al. 2021; Ahmad et al. 2022).

#### Sol-gel method

A highly efficient nanocatalyst was prepared using the sol-gel method by incorporating GG with nickel tungstate to create a homogeneous dispersion, followed by the addition of a ammonium persulfate (APS) solution. (Hussain et al. 2021). After stirring, a precipitate was obtained. This catalyst demonstrated great adsorption capacity for crystal violet and phloxine B

**Fig. 6:**
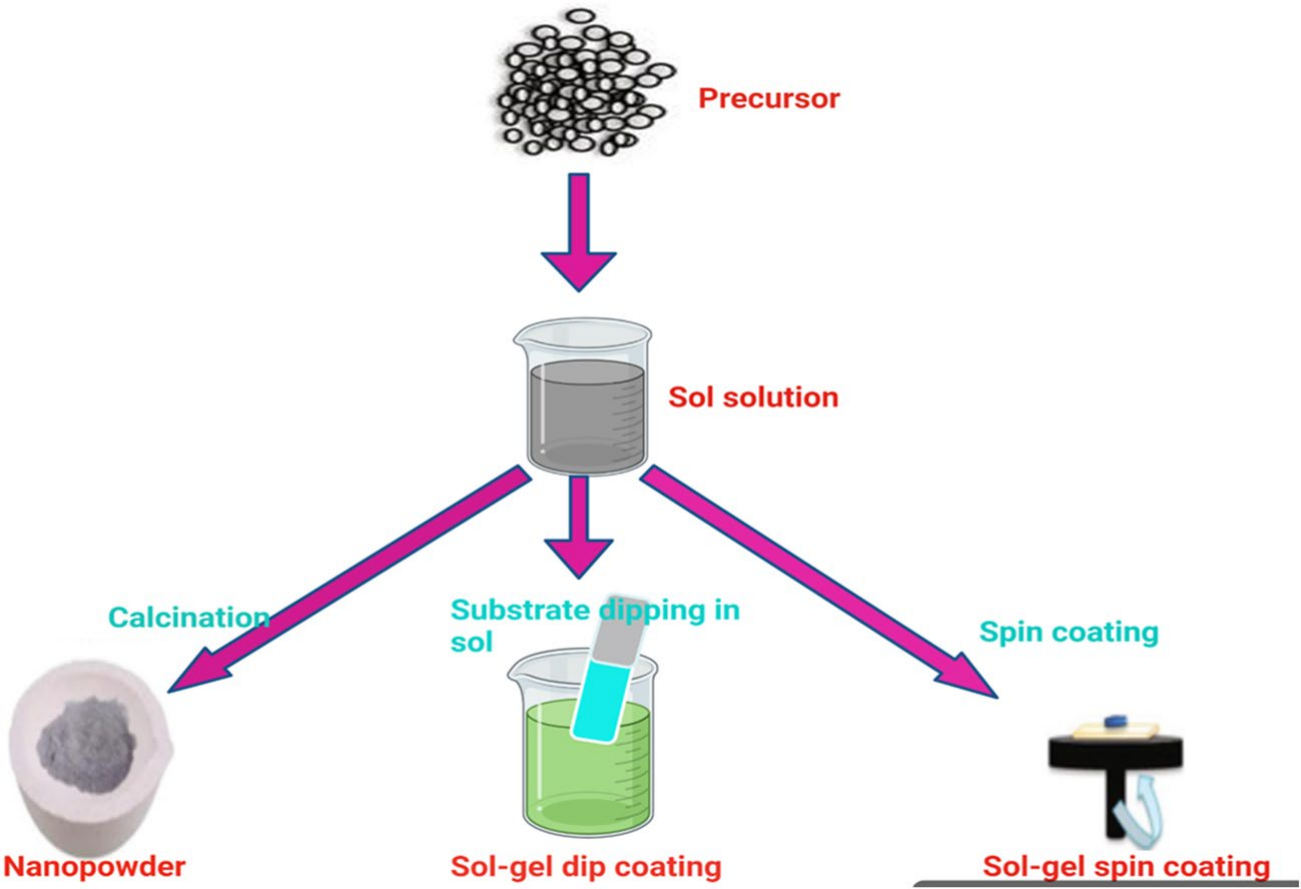
Two types of sol-gel technique

The sol-gel method is a widely-used technique for fabricating important catalysts, as shown in Fig. 6, which depicts two common variants: sol-gel dip coating and sol-gel spin coating. In the former, a substrate is immersed in a sol-gel solution, withdrawn at a controlled rate, and allowed to dry. While simple, this technique may not always produce uniform coatings over large areas. On the other hand, sol-gel spin coating involves spinning a substrate while dropping a sol-gel solution onto it, resulting in more precise and uniform coatings that can be as thin as a few nanometers. Overall, sol-gel spin coating offers a high level of control over coating thickness and uniformity, making it a valuable tool for catalyst fabrication.

This method was used to prepare a GG cerium tungstate nanocomposite (GG/CTNC), which has an ion exchange capacity of 1.30 mequiv g^−1^, making it effective for removing methylene blue (a cationic dye) and heavy metal ions from aqueous solutions (Gupta et al. 2014). In order to prepare the nanocomposite, a solution of cerium ammonium nitrate and a solution of sodium tungstate were gradually at pH 2, forming cerium tungstate precipitate, which was later mixed with a solution of GG. The precipitate was treated with HNO_3_ solution to transform it into the H^+^ form.

### Mixed nanocomposites of GG

Nanomaterials based on GG offer a unique combination of both metals and polymers in the composite, making them promising materials for water treatment applications, especially in the removal of dyes. The synergistic effect of the metal and polymer components enhances the adsorption capacity and selectivity of the composite, making it highly efficient in the removal of various types of dyes from contaminated water. These nanocomposites exhibit great potential as adsorbents in water treatment, owing to their unique properties and characteristics.

#### Crosslinking method

A nanostructure composed of erbium oxide and polyacrylic amide with GG exhibits excellent adsorptive behavior towards Nile blue dye. The crosslinking mechanism using a cross-linker such as MBA was employed to prepare the GG-polyacrylamide/erbium oxide nanocomposite Te GGPAAm/Er_2_O_3_ nanocomposite (Hussain et al. 2022). GG and acrylamide were crosslinked in the presence of Er_2_O_3_ and MBA, used as a cross-linker. A homogeneous mixture of acrylamide solution and an aqueous suspension of GG was obtained before adding APS as an initiator and Er_2_O_3_ as a filler.

#### Free radical polymerization

A novel nanocomposite consisting of a copolymer of polyacrylic acid with GG attached to silver nanoparticles (AgNPs/GG/Poly(AA)) was prepared using a two-step method (Singh and Dhaliwal 2021). A graft copolymer of polyacrylic acid with GG (poly(AA)/GG) was dissolved in an aqueous silver nitrate solution. The prepared copolymer was impregnated and crosslinked with silver metal nanoparticles in situ. A solution of sodium borohydride was added followed by MBA for crosslinking. The attachment of silver nanoparticles increased the surface area and adsorption capacity, resulting in a nanocomposite with excellent adsorption capability (833.33 mg g^−1^) for methylene blue.

#### Sonication

In the same way, the sonication method was used to synthesized nanocomposites by incorporating silver nanoparticles with polyester, immersed in the casting solution of UiO-66 filler, polyvinylidene fluoride (PVDF), GG, and ethylenediamine. UiO-66 is a Zr-containing MOF created by linking zirconium clusters with bridging ligands of terephthalic acid and modified dicarboxylate linkers. The final nanocomposite (PF/Ag/PVDF-GG/UiO-66) was obtained as a thin film polymeric membrane and demonstrated remarkable efficiency in removing CTAB and Congo red (Mofradi et al. 2021).

Two different biodegradable hydrogel-containing nanocomposites were obtained by embedding GG with acrylic acid and itaconic acid using microwave initiation to form GG-cl-poly(AA) and GG-cl-poly(IA-ipn-ANI), respectively. Both nanocomposites demonstrated excellent adsorptive behavior towards methylene blue (Choudhary et al. 2020). A GG-grafted acrylic acid GG/AA nanocomposite can also be prepared by silane modified nanoclay via a microwave irradiation method.

#### Mixing

A novel nanomaterial consisting of carboxyl methyl cellulose (CMC) and GG decorated with copper oxide nanoparticles (CuO NPs) was successfully prepared and utilized for the adsorption of malachite green dye from polluted water. To prepare the nanocomposite, dried CMC hydrogel was mixed deionized water at 700 rpm. Then GG was added to the hydrogel mixture, followed by gradual addition of CuO NPs, with different weight percentages (1, 3 and 5 wt%) (Naeini et al. 2021).

## Adsorption of metal ions from water

GG has been successfully used for the removal of Pb(II) from polluted water. Aqueous solutions of GG were found to remove up to 83% of lead when the initial concentration was 15 mg/L (Pal et al. 2014).

Also GG nanocomposites, prepared by different methods, were used for the removal of ions from waste water, as shown in the following sections. Table 2 presents some examples. The most important ones and their major preparation methods are listed in the following sections.

**Table 2:** Several nanomaterials obtained by different synthesis methods used in the removal of diverse ions from water

Nanomaterial	Synthesis	Adsorption	Adsorption capacity (mg g^−1^)/removal efficiency (%)	References
GG/bentonite	Solution intercalation	Pb(II)	97.94 mgg^−1^	Ahmad and Mirza (2018)
GG/XG/IC (XG-xanthan gum; IC-iota-carrageenan)	Stirring	Europium	44%	Levy-Ontman et al. (2022)
GG/XG	Photo crosslinking	Pb(II)	98.4mg g^−1^	Pal et al. (2017)
Semi IPN (GG/XG/PAA)	Irradiation of mixture	Pb(II) and Hg(II)	93% and 72%	Pal et al. (2020)
Groundnut husk modified with GG	Mixing and heating	Pb(II) Cu(II) and Ni(II)	9.76 mg g^−1^, 9.26 mg g^−1^, and 6.74 mg g^−1^, respectively	Ahmad and Haseeb (2015)
GHBA	Stirring and filtration	Pb(II) Cu(II) Zn(II) and Cd(II)	95.38%, 98.05%, 98.48%, and 97.56%, respectively	Singh and Kumawat (2013)
GG/PAm/PAA	Microwave irradiated	Hg	95%	Gihar et al. (2021)
GSA	Simple mixing	Zn(II), Cu(II), Cd(II), Pb(II), and Fe(II)		Singh and Sharma (2011)
GG/sodium lignosulfonate	Sonication	Cu and Co	709 mg g^−1^ and 601 mg g^−1^, respectively	Singh and Dhaliwal (2021)
GG/KPS/AA/EDTA	Free radical polymerization	Cd and Cu	99 mg g^−1^ and 90.3 mg g^−1^, respectively	Mubark et al. (2022)
AGG/salicylhydrazine	Mixing and heating	Cr(II), Co(II), and Ni(II)	1272.4 mg g^−1^, 748.86 mg g^−1^, and 521.81 mg g^−1^, remarkably	Ma et al. (2019)
GNTAA	Stirring and filtration	Fe(II), Pb(II), Cu(II), Zn(II), and Cd(II)		Singh et al. (2012)
GLF-BNCs	Ultrasound irradiated	Cd (II) and Cr (VI)	258 mg g^−1^	Dinari and Tabatabaeian (2018)
GG/nZnO	Sol-gel	Cr (VI)	98.63%	Khan et al. (2017)
Silica-GG-g-PAm	Dehyroxylation condensation	Cd(II)	99.4%	Singh et al. (2009)
Poly(AA-co-MMASH)	Condensation	Cu^+2^	196 mg g^−1^	Wen et al. (2022)
Phosphorylated GG/chitosan	Co-precipitation	Uranium and neodymium		Hamza et al. (2021)

### GG-based nanocomposites of natural and synthetic polymers

#### Solution intercalation method

Bentonite, an aluminum phyllosilicate clay composed mainly of montmorillonite, can be functionalized with GG to form an eco-friendly and sustainable adsorbent (GG/bentonite) for the efficient removal of Pb(II) from waste water (Ahmad and Mirza 2018). The nanocomposite was prepared using the solution intercalation method, consisting in adding bentonite to double-distilled and then combined it with GG solution.

A nanocatalyst made of GG, xanthan gum, and iota-carrageenan was an effective adsorbent for removing europium ions from waste water. The ratio of xanthan gum, GG, and iota-carrageenan in the fabricated nanocomposite was almost equal (Levy-Ontman et al. 2022).

#### Photo-crosslinked method

Likewise, nanostructures obtained by crosslinking xanthan gum and GG using UV irradiation were an effective adsorbent for Pb(II). In order to prepare the material, dry GG and xanthan gum were mixed with sodium lauryl sulfate and boron phosphide. A viscous slurry was formed by adding a small amount of double-filtered water. The slurry was then irradiated with UV under a nitrogen atmosphere for different periods. The addition of surfactants intimately mixed the gums (hydrophilic) and boron phosphide (hydrophobic) and increased the likelihood of crosslinking during the process. The radicals were then crosslinked to form the nanocomposite (Pal et al. 2017).

#### Irradiation method

An elastic semi-interpenetrating network of xanthan gum, GG, and poly acrylic acid (PAA) was designed using a similar strategy for the efficient removal of Pb(II) and Hg(II) from waste water (Pal et al. 2020). In order to prepare the adsorbent, a specific amount of sodium lauryl sulfate was added to GG and XG, and the mixture was thoroughly mixed and warmed to form a thick slurry. The prescribed amount of PAA and boron phosphide (BP) was added to the slurry, and the entire mass was homogenized and exposed to UV radiation.

#### Simple mixing

Another nanomaterial was synthesized by combination of ground nutshell powder with GG and polyvinyl acetate (PVA) as a solidifying agent. The material was tested for adsorption of nickel (II), lead (II), and copper (II) ions (Ahmad and Haseeb 2015). The biocomposite was prepared by dissolving ground nutshell powder in a solution of 3% GG powder. The PVA was then added as a solidifying agent.

The adsorption capacity of GG 4-hydroxybenzoic acid (GHBA) resin for metal ions such as Zn(II), Cu(II), Pb(II), and Cd(II) was determined based on their distribution coefficient value by column chromatography at different pH values. The removal (%) of metal ions increased initially upon pH increase, then decreased. The optimal pH range for maximum adsorption was 4–6 (Singh and Kumawat 2013; Saya et al. 2021). To synthesize the GHBA resin, GG was allowed to react with p-hydroxybenzoic acid. The resulting product was vacuum-filtered and washed with aqueous methanol containing a few drops of HCl to remove any inorganic impurities and neutralize excess NaOH. Finally, the resin was washed with methanol and dried.

### Microwave-assisted technique

**Fig. 7:**
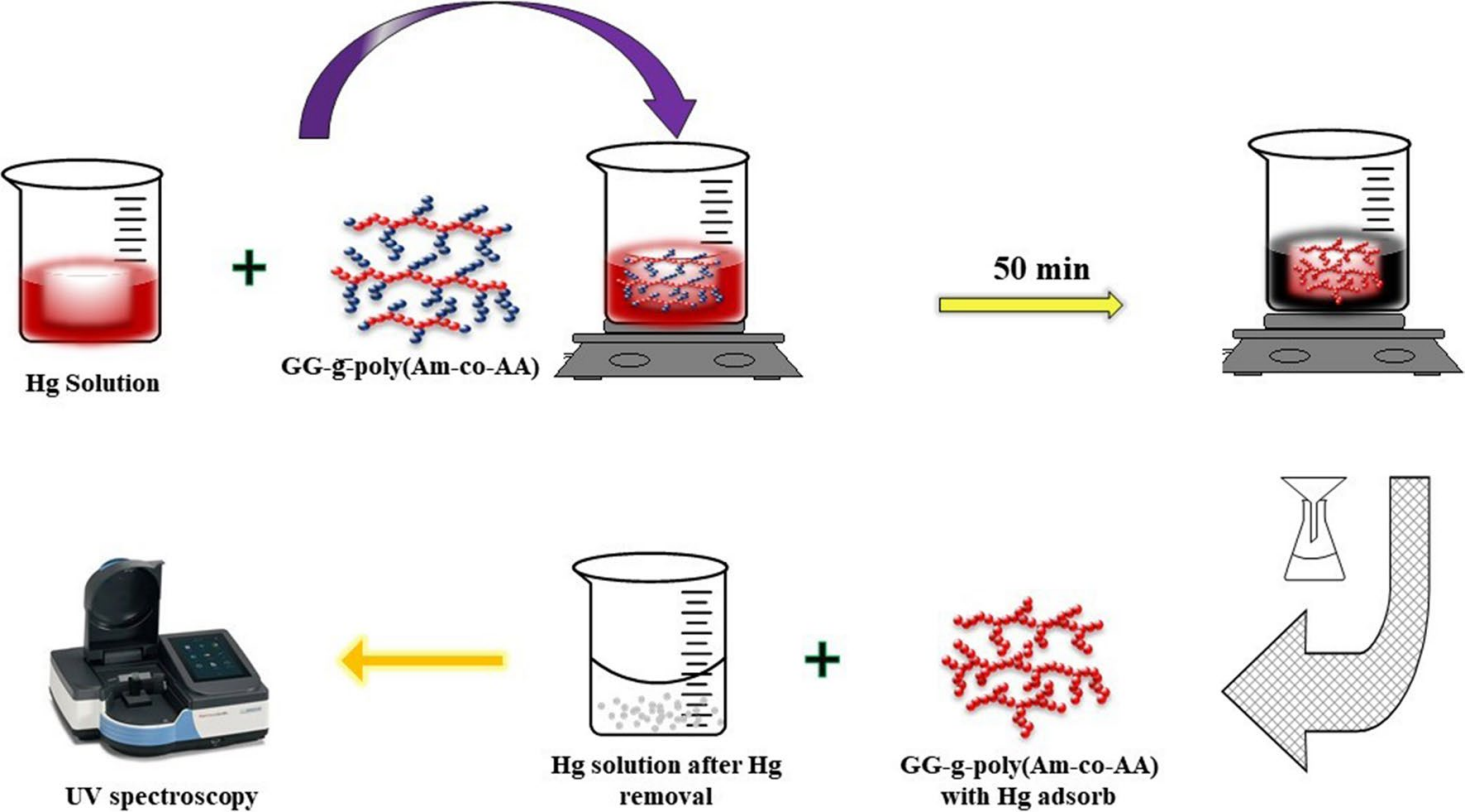
Preparation of GG-g-Poly(Am-co-AA) nanocomposite by a microwave-assisted technique (redrawn from (Gihar et al. 2021))

Similarly, a GG nanocomposite with poly acryl amide and acrylic acid was synthesized for the adsorption of heavy toxic metal ions of mercury, as shown in Fig. 7, using microwave-assisted technique.

GG-g-poly(Am-co-AA) was obtained by dissolving GG in double-distilled water. Am and AA were put into the GG solution and the homogenous mixture was exposed to microwave radiation in a home microwave for 120 s at 80% power before being stopped by mixing a saturated solution of hydroquinone (Gihar et al. 2021). The use of a microwave-assisted technique allows for a faster and more efficient synthesis compared to traditional methods.

### GG-based nanocomposites of the main group elements

#### Sonication

GG-based sodium ligno sulphonate nanocomposite was synthesized by a sonication method and used for adsorption of copper and cobalt ions from waste water. AA was added to a solution of KOH, and the mixture was used to dissolve GG. Sodium ligno sulphonate, ammonium persulfate and *N,N*-methylenebisacrylamide (NMBA) were also added and went through ultrasonication to finish the gelation process (Singh and Dhaliwal 2021).

#### Free radical polymerization

A nanomaterial based on GG was synthesized by a free radical polymerization mechanism and used for the adsorption of copper and cadmium ions from water. To create the hydrogel, GG was dissolved in distilled water and mixed with a combination of acrylamide and acrylic acid (Am-AA). KPS dissolved in water was added, then MBA) in distilled water. The water bath was maintained at 70 °C to complete the polymerization and produce the hydrogel, GG-g-(Am-co-AA) (GGH) (Mubark et al. 2022).

#### Co-precipitation

GG was grafted with salicylhydrazine to produce a hydrophobic nanocomposite with a large surface area. The adsorption capacity of the nanocomposite was 521.81 mg/g, 748.86 mg/g, and 1272.4 mg/g for cobalt (II), chromium (II), and nickel (II), respectively. To prepare the nanocomposite, AGG was dissolved in ethanol and was mixed with salicylhydrazine and p-toluenesulfonic acid (Ma et al. 2019).

GG-based nanocomposites with sulfonic acid were synthesized to remove different heavy metal ions at pH 7. The adsorption capability of the nanocomposites showed the following order: Cu(II) < Fe(II) < Zn(II)< Cd(II) < Pb(II). The distribution coefficient values for Zn(II), Pb(II), Cu(II), Cd(II), and Fe(II) were 4.215, 6.06, 4.25, 2.20, and 5.40, respectively. To fabricate the GG powder-based cation exchanger, it was mixed with sodium 1,2-epoxypropyl sulphonate, which was produced by the reaction of sodium bisulphite (NaHSO_3_) and epoxychloropropane (Singh and Sharma 2011).

### GG-based nanocomposites of transition, lanthanide, and actinide elements

#### Ultrasound irradiation

The study demonstrated the efficacy of using a green strategy for synthesizing Fe_3_O_4_ layered double hydroxide (LDH) at GG nanomaterial (Fe_3_O_4_@ layered double hydroxide@ GG bionanocomposites, i.e., GLF-BNCs) through an in situ embedding procedure, which resulted in the development of electrostatically bound (Ni-Al) LDH layered Fe_3_O_4_ nanoparticles. This nanomaterial was highly effective in removing Cd(II) and Cr(VI) ions from water.

GG was combined with distilled and ethanol in a 4:2 molar ratio and magnetic LDH was then added to the reaction mixture in varying doses (0.01 g, 0.02 g, and 0.04 g), resulting in bionanocomposites with 2%, 4%, and 8% incorporation, respectively. The reaction solution was refluxed in nitrogen and subjected to ultrasound irradiation (Dinari and Tabatabaeian 2018).

#### Sol-gel

The use of GG-based ZnO nanocomposite (GG/nZnO) was an effective method for extracting Cr(VI) ions from polluted water. The composite was synthesized by mixing GG powder with mixture of isopropyl alcohol and distilled water (3:1 ratio) until a viscous gel was formed. Then zinc acetate was added and also a NaOH solution in isopropyl alcohol (20 mL) to convert the zinc acetate to ZnO nanoparticles, which were then fixed onto the polymeric guar mesh (Khan et al. 2017).

### Mixed nanocomposites of GG

#### Condensation

A highly efficient adsorbent, GG-based silica/polyacrylamide nanocomposite, was synthesized through the condensation of tetraethyly orthosilicate with GG-based polyacrylamide. This material demonstrated excellent Cd(II) adsorption capacity (2000 mg/g) with good reusability and regeneration even after 10 cycles, as confirmed by Langmuir’s adsorption isotherm. To prepare the nanocomposite, GG-g-PAm was dissolved in distilled water, while tetraethyly orthosilicate was dissolved in ethanol. The two solutions were then combined with a third solution containing ammonium hydroxide (Singh et al. 2009).

Another nanocatalyst with excellent adsorptive behavior towards Cu^2+^ ions was prepared by modifying dialdehyde GG (DAG) with polymethyl methacrylate through condensation with polyhydrazide. DAG was prepared using a documented process, and it was mixed with poly (AA-co-MMASH) and acetic acid (1 mL). A bright yellow solid product GSA was obtained (Wen et al. 2022).

#### Co-precipitation

A nanomaterial that shows great potential for wastewater remediation is phosphorylated GG (PGG) and chitosan composite, which exhibits strong adsorption capabilities for uranium and neodymium ions. The synthesis of this nanocomposite involved adding chitosan, an aqueous solution of acetic acid, followed by the addition of PGG powder once the chitosan had completely dissolved. Afterwards, epichlorohydrin diluted in dioxane was added, and pH adjustment to 9 was made with NaOH solution. A light brown precipitate, denoted as PGG@C, was obtained (Hamza et al. 2021).

## Physiochemical techniques for characterization

Several techniques can be used for characterization of nanocomposites used for as adsorbents. Some examples are referred in the following sections.

### Thermal analysis

**Fig. 8:**
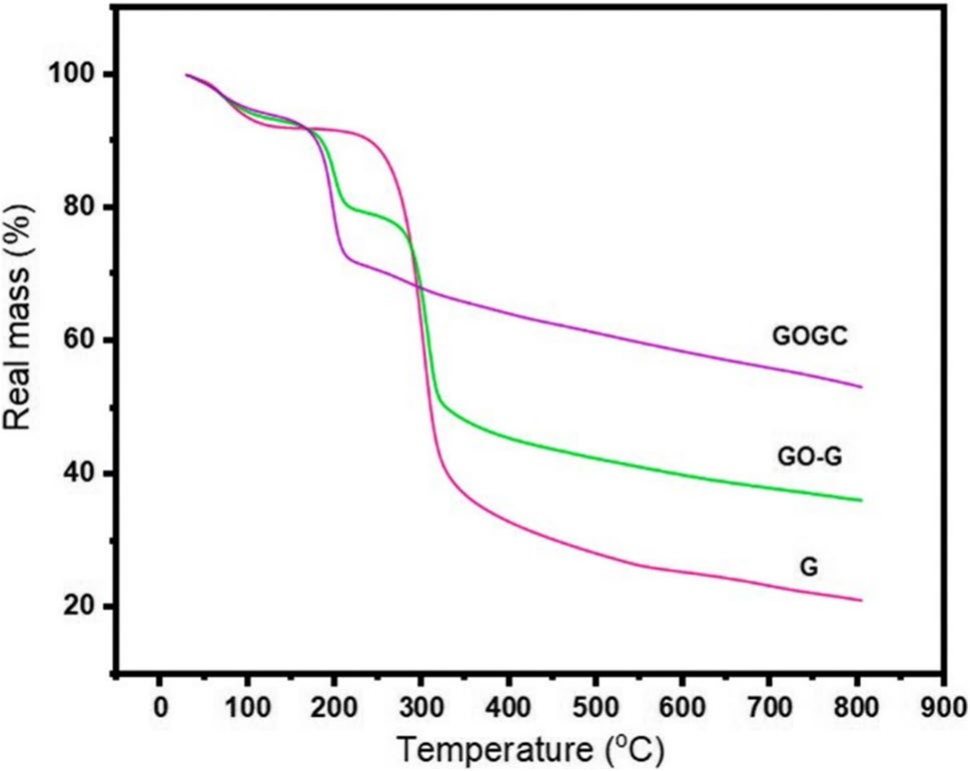
TGA curves of graphene oxide/guar gum/CuO (reprinted from (Akbarzadeh et al. 2023) with permission from Elsevier)

The thermogravimetric analysis (TGA) is a useful technique to study the chemical and physical modifications of adsorbents over time and temperature. TGA curves can provide information about the thermal stability of the nanocomposite materials, including the weight loss and the temperature at which it occurs (Hu et al. 2016). The analysis of the TGA curves allows to determine the strength of the sample material and understand how physically attached water evaporates with increasing temperature. TGA curves can also be used to compare the thermal stabilities of different adsorbents, as well as their precursors. Additionally, weight loss data as a function of temperature can provide insight into possible reactions, such as the pyrolysis of oxygen-containing functional groups under heating conditions. Therefore, TGA is an important tool for understanding the thermal properties and stability of adsorbents, which is crucial for their efficient use in different applications. The thermal properties of as synthesized graphene oxide/guar gum/CuO were analyzed through TGA curves, as shown in Fig. 8.

### Morphological analysis

**Fig. 9:**
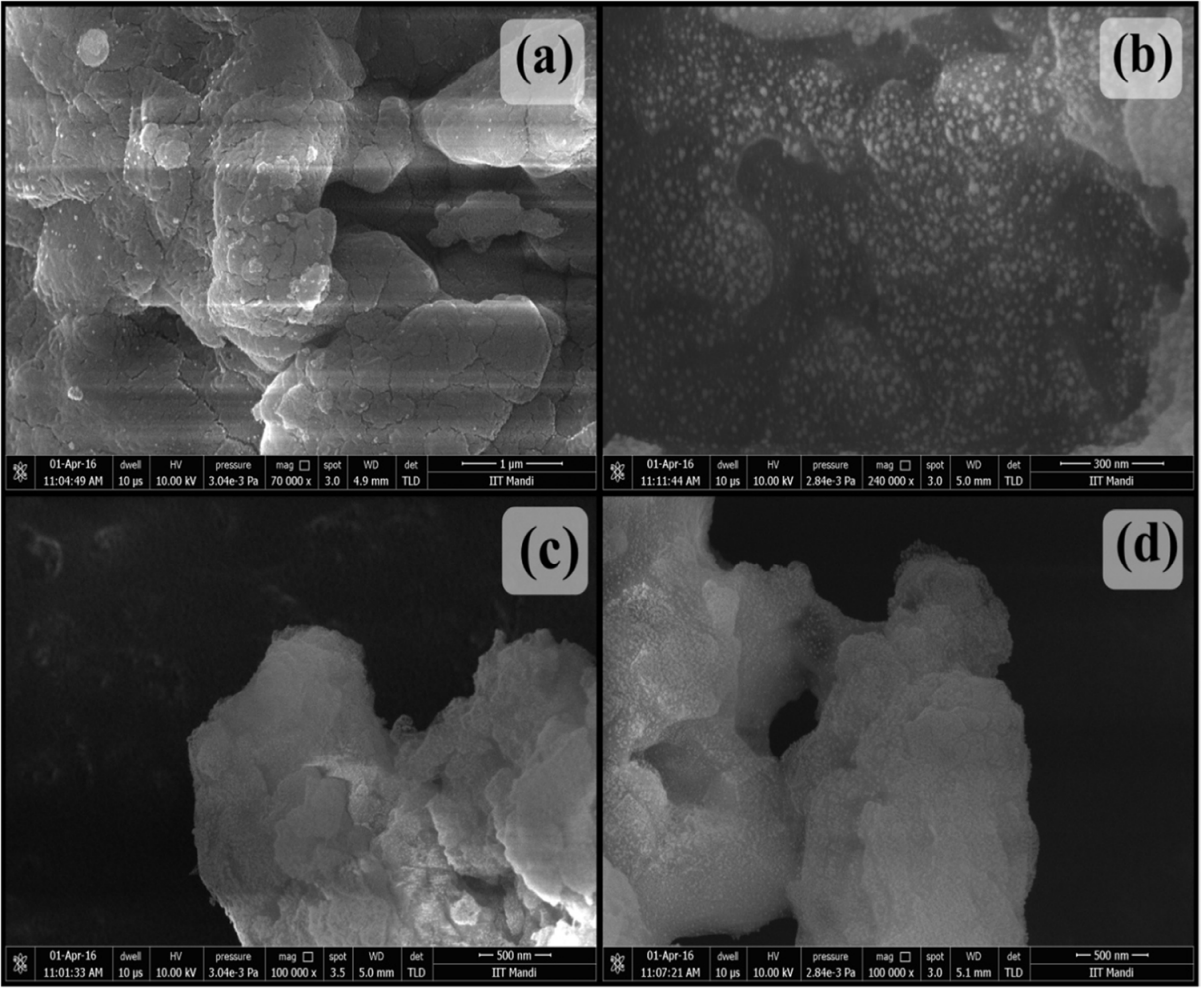
SEM images of Pc-cl-GG/SPION at different magnification (reprinted from (Sharma et al. 2017) with permission from Elsevier)

Advanced imaging techniques such as scanning electron microscopy (SEM), field emission scanning electron microscopy (FESEM), transmission electron microscopy (TEM), and high resolution transmission electron microscopy (HRTEM) provide valuable insights into the surface and internal morphology of adsorbents at different scales and wavelengths (Zhang et al. 2021). Particle size distribution histograms can be generated by analyzing various grid regions, allowing the confirmation of successful synthesis or incorporation of desired nanoparticles (Zhu et al. 2020). Additionally, atomic force microscopy (AFM) allows to examine the external topography of nanocomposites in 2D and 3D. By comparing AFM images of the nanomaterial sample before and after adsorption, useful information about the interlinked adsorbent and adsorbate can be obtained. SEM images of Pc-cl-GG/SPION at different magnifications are shown in Fig. 9. These advanced imaging techniques are essential in providing a comprehensive understanding of the structure and behavior of nanocomposites for effective wastewater remediation.

### Surface charge analysis

The surface charge density of an adsorbent can be studied using the zeta potential technique. By analyzing the deviation of zeta potential values as a function of pH, valuable information can be obtained about the change in surface charge density with respect to pH, which can help understanding the adsorption mechanism. It is important to note that the variation of functional groups in the adsorbent and in the dye is determined by their respective polarities (Saya et al. 2021).

### Elemental analysis

This is a powerful tool that allows both qualitative and quantitative analysis of specific elements in a material. This technique can provide valuable information about the chemical composition of the material being studied. For example, Singh et al. used an elemental analyzer to investigate the composition of GHBA resin and GG-based nanocomposites. By measuring the amount of carbon, hydrogen, and oxygen, they were able to confirm the theoretical values of 53.32%, 5.22%, and 40.05%, respectively. (Singh and Kumawat 2013).

### Vibrating sample magnetometry (VSM)

**Fig. 10:**
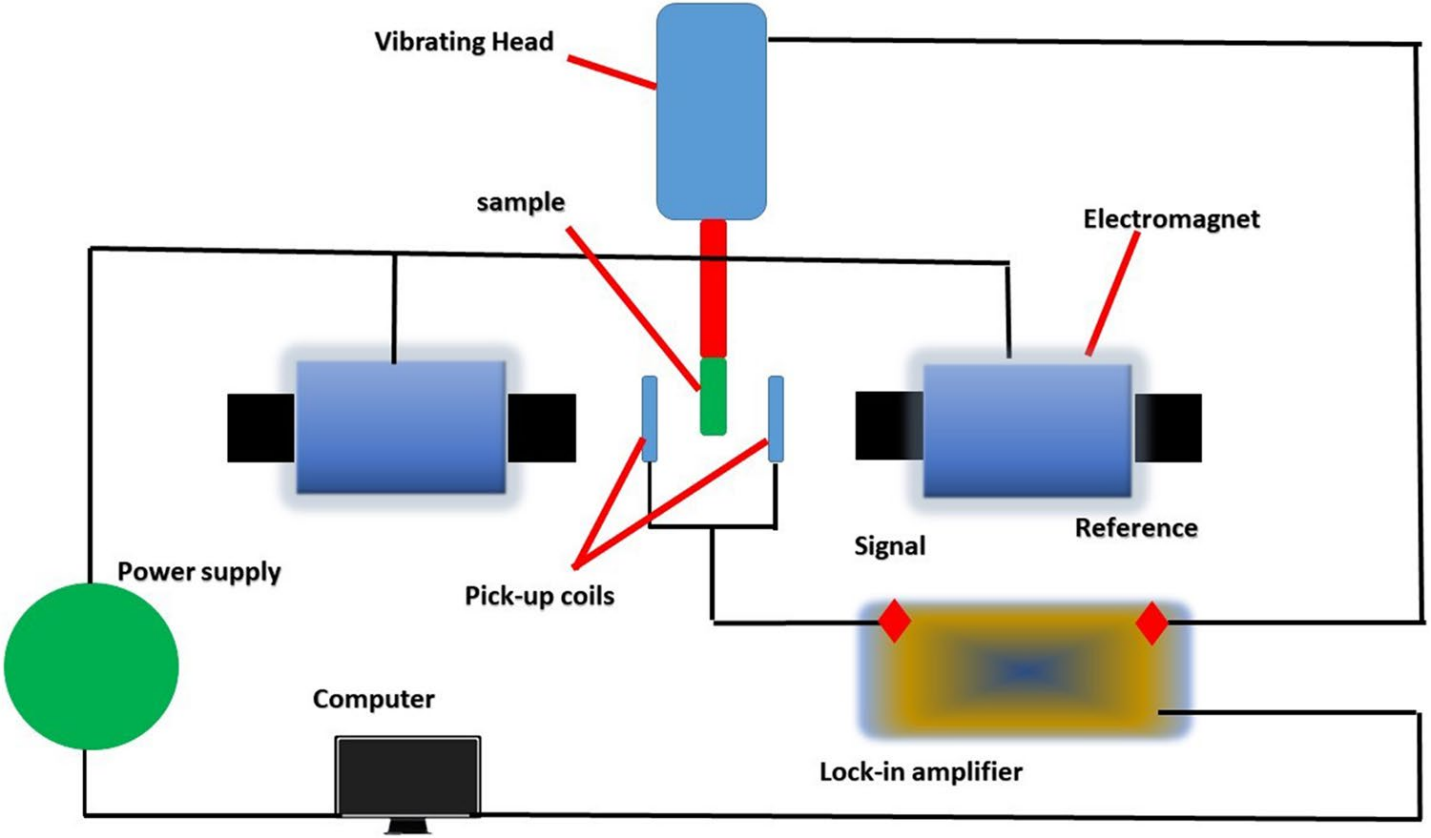
Vibrating sample magnetometer and its main components

To measure the magnetic properties of a material, a VSM (vibrating sample magnetometer) instrument is commonly used, particularly when the material is expected to exhibit magnetic behavior due to the incorporation of magnetic components during preparation (Yildiz et al. 2020). By determining the magnetic moment of the material, a VSM can provide valuable insights into the material’s magnetic properties and potential applications. Figure 10 depicts a VSM instrument and its essential components. The instrument includes an electromagnet for producing a magnetic field, pick-up coils that detect changes in the magnetic field after interacting with the sample, a sample holder that holds the sample in the magnetic field, a power supply to generate the magnetic field, a lock-in amplifier that amplifies signals received by the pick-up coils, and a feedback system that receives data from the lock-in amplifier and stores it for further analysis. Understanding how the VSM instrument works is crucial for accurately measuring a material’s magnetic properties and unlocking its potential applications.

### Spectroscopic analysis

Various spectroscopic techniques are utilized to comprehensively characterize the synthesized nanocomposites and investigate the structural properties, performance, and inter-relationships, particularly in adsorption/desorption and photodegradation phenomena.

Fourier transform infrared (FTIR) spectroscopy is a powerful technique to confirm the presence of specific functional groups attached to the adsorbent and the existence of hydrogen bonding in the system (Wei et al. 2022). FTIR spectra are also useful to determine if adsorption or degradation are occurring. In addition, the disappearance of peaks in the FTIR spectra after reaction confirms the degradation of the dye to simpler compounds.

**Fig. 11:**
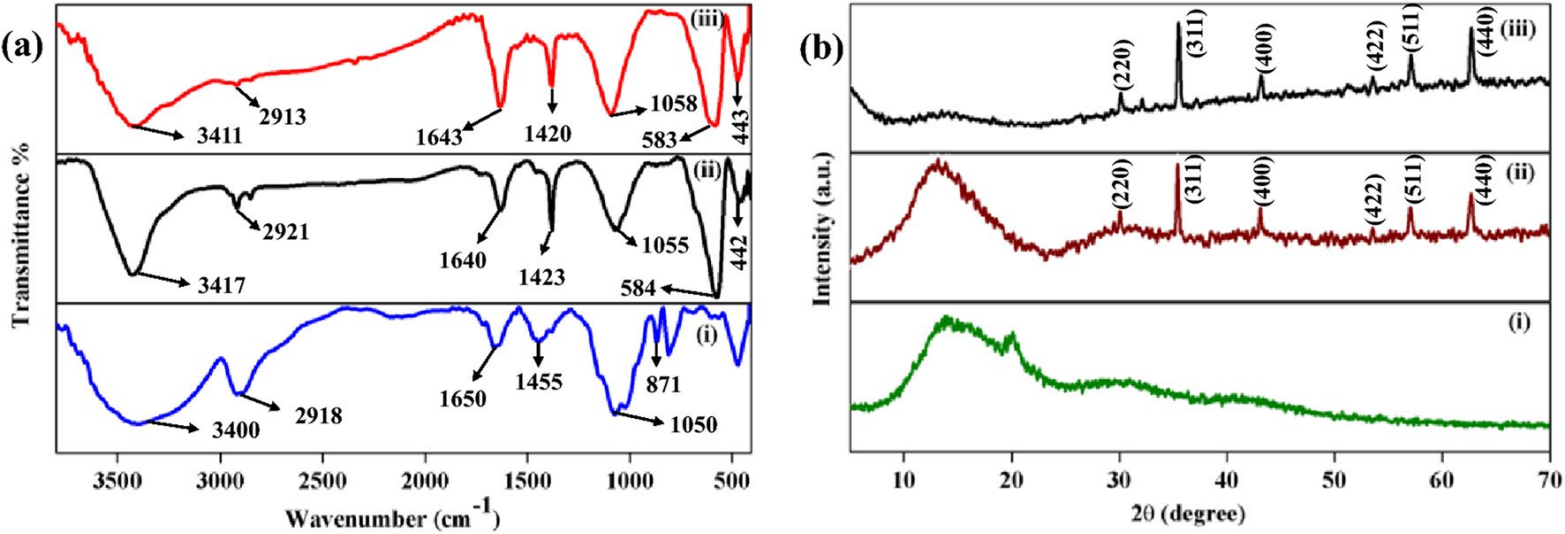
**a** FTIR spectra of (i) guar gum, (ii) GG-Fe_3_O_4_, and (iii) reused GG-Fe_3_O_4_; **b** PXRD of (i) GG, (ii) GG-Fe_3_O_4_, and (iii) reused GG-Fe_3_O_4_ (reprinted from (Kumar et al. 2022b) with permission from Springer)

Powdered X-ray diffractive (PXRD) analysis is beneficial to verify the composition and structure of crystalline materials. The appearance or disappearance of different peaks at different 2θ values provides crucial data regarding the crystal planes and confirms the interaction of specific molecules. During encapsulation of Fe_3_O_4_ particles on guar gum, structural and phase structural changes investigated by FTIR and PXRD are shown as Fig. 11 (Kumar et al. 2022a).

The X-ray photoelectron spectroscopy (XPS) is used to understand the chemical composition of a material and analyze the synthesized adsorbents in greater detail (Aziz and Ismail 2017). Nuclear magnetic resonance (NMR) spectroscopy is also widely used to analyze the synthesized adsorbents. NMR and XPS can be used to better understand the adsorption process and the quality of the adsorption forces at work. UV-Vis spectroscopy is another effective method to analyze the electronic configuration of samples, particularly those containing aromatic compounds or a broad range of UV-Vis active compounds (Bauer et al. 2021).

## Conclusions

Nanocomposites are an exciting and promising field of research for the development of efficient and effective adsorbents for wastewater treatment. This review explains the significance of gum-based nanomaterials in wastewater treatment, including preparative steps, characterization techniques, kinetics models, and the degradation and adsorption mechanisms involved.

Various techniques such as SEM, TEM, FTIR, XRD, and NMR spectroscopy can be used to characterize the synthesized nanocomposites and gain insights into their structure, morphology, and chemical composition. Thermogravimetric analysis (TGA) and zeta potential techniques can provide important data on the thermal strength and surface charge density of adsorbents, respectively, which can aid in the development of reasonable adsorption mechanisms. The use of advanced characterization techniques such as VSM and XPS can also help in understanding the magnetic and chemical properties of synthesized adsorbents. Using these various techniques, researchers can gain insights into the structural-property-performance inter-relationships of nanocomposites, particularly in adsorption/desorption and photodegradation phenomena. Ultimately, the use of these characterization techniques is essential in the development of effective adsorbents for wastewater treatment and will continue to be an important area of research in the future.

## Data Availability

Data will be made available upon request
